# Determinants of exclusive breastfeeding in infants of six months and below in Malawi: a cross sectional study

**DOI:** 10.1186/s12884-020-03160-y

**Published:** 2020-08-17

**Authors:** Yusuf M. Salim, William Stones

**Affiliations:** 1 Karonga District Hospital, Private Bag 1, Karonga, Malawi; 2 College of Medicine, Private Bag 360, Blantyre, Malawi

## Abstract

**Background:**

UNICEF and WHO recommend that all children should be exclusively breastfed for the first 6 months of life as breastmilk contains all the nutrients an infant needs during this period. In Malawi, exclusive breastfeeding has been declining from 72% (2009), 70.2% (2014) and 61% in the most recent survey (2015–16). We aimed to determine factors associated with exclusive breastfeeding in Malawi.

**Methods:**

We used data from the Malawi Demographic and Health Survey (MDHS) 2015–2016. Survey records for 2059 mothers of children aged 6 months and below were identified and potential factors influencing infant feeding were examined. Logistic regression analysis was carried out to model determinants of exclusive breastfeeding (EBF).

**Results:**

EBF declined in proportion to the age of the infant. Significant associations with continuing EBF were age of the mother, ethnicity of the mother, sex of infant and number of siblings. Members of the Tumbuka (OR = 1.71, CI. 1.13–2.59) and Ngoni (OR = 2.05, CI. 1.38–3.05) communities were more likely to practice EBF. In addition, mothers with female babies (OR = 1.35, CI. 1.08–1.70) and those with 3–4 children (OR = 1.47, CI. 1.04–2.08) were more likely to engage in EBF.

**Conclusion:**

We identify important variations in EBF practices among population sub-groups in Malawi that need to be considered when framing health education messaging. Work is needed to assess the impact of more targeted messaging, whether delivered via ‘ten steps’ to successful breastfeeding under Baby Friendly Hospital Initiative (BFHI) programming or other health education and awareness campaigns to sensitize communities on implications of some cultural practices on the lives of babies. The potential role for mass media, targeted Health Surveillance Assistants’ (HSA) home visits and male involvement also require exploration.

## Background

Exclusive breastfeeding under 6 months of age is defined as the proportion of children, 0–6 months of age, fed only breastmilk, with the exception of oral rehydration solution, vitamins, minerals, and/or medicines [1]. UNICEF and WHO recommend that children should be exclusively breastfed for the first 6 months of life to achieve optimal growth, development and health [2]. This implies that no other foods or liquids are provided, including water.

Breast milk is a safe and nutritive diet for the healthy growth and development of infants. It also provides economic benefits by reducing both the direct and indirect costs related to healthcare and infant feeding [3].

Improving breastfeeding practices could save the lives of more than 800,000 children under 5 every year, the vast majority of whom are under 6 months of age [2]. Exclusive breastfeeding confers both short and the long-term benefits to both the child and mother. Among others breastmilk contains all the nutrients an infant needs in the first 6 months of life. It is the most ideal, safe, and complete food that a mother can provide for her newborn [4].

Exclusive breastfeeding for the first 6 months of life improves the growth, health and survival status of newborns and is one of the most natural and best forms of preventive medicine [5]. In addition, exclusive breastfeeding plays a pivotal role in determining the optimal health and development of infants, and is associated with a decreased risk for many early-life diseases and conditions, including otitis media, respiratory tract infection, diarrhea and early childhood obesity [5].

The 2017 USAID Report on addressing barriers to exclusive breastfeeding reveals that in Malawi, the prevalence of early introduction of foods and liquids in the first 6 months is at 34.6% [6]. This implies that 65.4% of the infants are exclusively breastfed. In addition to that, MDHS 2015–2016 claims that 61% of children under 6 months are exclusively breastfeed compared to exclusive breastfeeding prevalence of 72% reported in 2009–2010 MHDS [7].

Furthermore, the MICS Survey (Millenium Development Goals Endline Survey) undertaken in 2014 found the prevalence of exclusive breastfeeding to be at 70.2% [8]. This trend depicts suboptimal performance in exclusive breastfeeding which remains below the WHO/UNICEF recommendation of 90% for developing countries [9].

A more detailed understanding of the factors associated with exclusive breastfeeding in Malawi is needed to develop effective interventions to improve these rates and thus improve health and wellbeing of infants. The purpose of the present study is to use existing representative MDHS data to identify the factors associated with exclusive breastfeeding in Malawi. The secondary data analysis aims to investigate the factors associated with rates of exclusive breastfeeding after controlling for individual, household and community characteristics.

Malawians are part of the large Bantu population that migrated northward from South Africa at around the turn of twentieth century [10]. The major ethnic groups found in Malawi are Chewa, Nyanja, Tumbuka, Yao, Lomwe, Sena, Tonga, Ngoni and Ngonde. Chewa are the most numerous having 34.3% of the whole population [11]. They are predominantly concentrated within the central region of Malawi. The Yao tribe is mostly found in southern lakeshore of Lake Malawi.

Tumbuka is the main tribe found in the northern part of the country, while Tonga dominate the northern lakeshore district of Nkhatabay. The Lomwe tribe is found in the southern part of Malawi particularly Mulanje and Thyolo Districts. Ngonde migrated from north of Africa and settled in the north of Malawi while Sena people came from Mozambique and settled along the lower Shire River in Chikwawa and Nsanje Districts [12]. The Ngoni tribe is concentrated in Ntcheu District – central Malawi, and Mzimba District – northern Malawi.

## Methods

We used data from the 2015–16 MDHS. The dependent variable was exclusive breastfeeding and the independent variables were the factors potentially affecting exclusive breastfeeding. We examined the influence of maternal determinants (age of mother, education of mother, religion of mother, employment status of mother, ethnicity of mother, region where mother resides, wealth index of mother and marital status), and infant determinants (sex of the baby, age of the baby and number of children born to the mother). These variables were assessed quantitatively to measure their significance in affecting exclusive breastfeeding.

The study used the data set which involved information on women with infants of 6 months and below. This data set was used because the women would have provided the most current information about feeding status and on the determinants of exclusive breastfeeding.

The MDHS was a nationally representative sample of 25,146 women aged 15–49 of whom 24,562 were interviewed during the survey, representing a response rate of 98%. The 2015–16 MDHS provides reliable estimates at the national and regional levels, for urban and rural areas, and for each of the 28 districts in Malawi. The survey methods are described in detail in the MDHS report [7].

After access to dataset was granted, the data were analyzed using statistical software (STATA Version 12 S/E) and bivariate tables generated. Logistic regression analysis was carried at two levels to identify factors associated with exclusive breastfeeding. Firstly, binary logistic regression analysis was carried out and variables with *p*-value < 0.05 were included in the final multivariable logistic regression analysis. Strength of association was measured using odds ratios and 95% confidence intervals. A *p*-value < 0.05 was set for statistical significance.

MDHS used sample weights to ensure accurate representation of the proportion of women at national and regional levels. Therefore, in this analysis, only the weighted numbers are shown in the tables.

The data used in this analysis were from woman’s questionnaire in which women with babies from 0 to 6 months were considered. A variable of exclusive breastfeeding was generated by considering those women who gave their children food other than breastmilk including water within the period of 6 months from birth of the child. Whether currently breastfeeding or not, a woman with child within age of 6 months and below was considered during the process of generating the variable of exclusive breastfeeding.

Permission to use the secondary data was sought from the Demographic and Health Surveys (DHS) Program and a waiver of ethical review was granted by the College of Medicine Research Ethics Committee (COMREC).

## Results

### Univariate analysis

This was done to describe the characteristics of the variables of interest in the study by calculating the frequencies and percentages of the categories for each explanatory variable. The dataset consisted of 17,286 women. After data cleaning the analysis of the survey data included 2059 mothers of children aged 6 months and below. The mean age of the women was 25.7, 95% CI (25.37–26.07).

### Breastfeeding practices among mothers

In this survey 1059 (51.43%) of children aged 6 months and below were exclusively breastfed and 1000 (48.57%) were not exclusively breastfed (Table 1).

**Table 1:** Breastfeeding practices of the respondents

**Feeding Practice**	**Frequency (n)**	**Percent (%)**
Exclusively breastfed	1059	51.4
Not exclusive breastfed	1000	48.6

### Social and demographic characteristics of study participants and their children

The social and demographic characteristics include age of mother, education of mother, religion of mother, region of mother, ethnicity of mother, occupation of mother, wealth index of mother, marital status of mother, sex of the infant and age of the infant.

About half of the mothers (1030) were between the ages 15 to 24 years, 1344 (65.3%) had primary level of education, 1139 (55.3%) were Christians, 1005 (48.8%) were from the southern region, 605 (29%) were of Chewa tribe, 1151 (55.9%) were employed, 466 (22.6%) were the poorest and 1742 (82.6%) were married. Most women, about 1064 (51.7%) had 1–2 children. Almost half of the children, 1032 (50.1%) were females and 873 (42.4%) children were aged between 0 to 2 months (Table 2).

**Table 2:** Characteristics of mothers and children

**Variables**	**Frequency (n)**	**Percent (%)**
**Age of mothers in years**		
15 to 24	1030	50.0
25 to 34	759	36.9
35 and above	270	13.1
**Education of mothers**		
No education	243	11.8
Primary	1344	65.3
Secondary	432	20.9
Higher	40	1.9
**Religion of mothers**		
Muslim	909	44.2
Christian	1139	55.3
Other	11	0.5
**Region of mothers**		
Northern region	380	18.5
Central region	674	32.7
Southern region	1005	48.8
**Ethnicity of mothers**		
Chewa	605	29.0
Tumbuka	222	10.8
Lomwe	336	16.3
Tonga	76	3.7
Yao	287	13.9
Sena	119	5.8
Nkhonde	18	0.9
Ngoni	237	11.5
Mang’anja	53	2.6
Nyanja	49	2.4
Other	57	2.8
**Employment status of mothers**		
Yes	1151	55.9
No	908	44.1
**Wealth index of mothers**		
Poorest	466	22.6
Poorer	454	22.1
Middle	390	18.9
Richer	379	18.4
Richest	370	17.9
**Marital status**		
Never in union	131	6.4
Married	1742	82.6
Widowed/Divorced	186	9.1
**Number of children of mothers**		
1–2	1064	51.7
3–4	613	29.8
5 and above	382	18.6
**Sex of Infants**		
Male	1027	49.9
Female	1032	50.1
**Age of infants**		
0–2 months	873	42.4
3–4 months	573	27.8
5–6 months	613	29.8

Note: Wealth category was derived based on data from the household’s ownership of consumer goods, dwelling characteristics, type of drinking water source, toilets facilities

### Feeding practices by age of child

Table 3 shows breastfeeding practices by child’s age. Only 15.2% of infants aged between 5 to 6 months are exclusively breastfed compared with 77.3% of infants aged between 0 to 2 months and 50.8% of infants aged between 3 to 4 months.

**Table 3:** Breastfeeding practices by age of child

**Feeding Practice**	**Age of Child**		
	***0–2 months***	***3–4 months***	***5–6 months***
Exclusively breastfed	675 (77.3%)	291 (50.8%)	93 (15.2%)
Not exclusively breastfed	198 (22.7%)	282 (49.2%)	520 (84.8%)

Bivariate analyses are shown in Table 4.

**Table 4:** Maternal and child characteristics and rates of exclusive breastfeeding infants of 6 months and below in Malawi (*n* = 2059)

**Variables**	**EBF n (%)**	**No EBF n (%)**	**Uncorrected Chi(2) Design based (F statistic)**	***P*-value**
**Age of mothers (years)**				
15 to 24	503 (24.6%)	531 (25.9%)	28.0603	
25 to 34	399 (19.5%)	356 (17.4%)	10.5424	< 0.0001
35 and above	173 (8.4%)	85 (4.2%)		
**Education of mothers**				
No education	143 (6.9%)	109 (5.3%)	7.3585	0.2063
Primary	683 (33.4%)	672 (32.8%)	1.5265	
Secondary	229 (11.2%)	180 (8.8%)		
Higher	20 (0.9%)	12 (0.6%)		
**Religion of mothers**				
Muslim	184 (9.0%)	147 (7.2%)	14.6223	
Christian	889 (43.3%)	823 (40.2%)	1.5587	0.1551
Other	4 (0.4%)	4 (0.2%)		
**Region of mothers**				
Northern region	117 (5.7%)	106 (5.2%)	0.8370	
Central region	419 (20.5%)	398 (19.4%)	0.3103	0.7222
Southern region	538 (26.3%)	468 (22.9%)		
**Employment status of mothers**				
Yes	485 (28.8%)	580 (28.4%)	4.8316	
No	589 (23.7%)	392 (19.2%)	2.9592	0.0858
**Ethnicity of mothers**				
Chewa	340 (16.6%)	364 (17.8%)		
Tumbuka	116 (5.7%)	78 (3.8%)		
Lomwe	178 (8.7%)	186 (9.1%)		
Tonga	18 (0.9%)	15 (0.8%)		
Yao	177 (8.7%)	151 (7.4%)	27.8252	0.0150
Sena	42 (2.1%)	43 (2.1%)	2.2981	
Nkhonde	6 (0.3%)	4 (0.2%)		
Ngoni	146 (7.1%)	78 (3.8%)		
Mang’anja	29 (1.4%)	30 (1.5%)		
Nyanja	11 (0.6%)	10 (0.5%)		
Other	11 (0.6%)	15 (0.7%)		
**Wealth index of mothers**				
Poorest	265 (12.92%)	247 (12.1%)		
poorer	224 (10.95%)	236 (11.5%)		
Middle	209 (10.21%)	179 (8.7%)	9.3469	0.1895
Richer	183 (8.93%)	177 (8.7%)	1.5405	
Richest	194 (9.48%)	134 (6.6%)		
**Marital status**				
Never in union	63 (3.1%)	73 (3.6%)	11.4396	0.1786
Married	930 (45.4%)	800 (39.1%)	1.5309	
Widowed/Divorced	82 (4.0%)	99 (4.9%)		
**Sex of Infants**				
Male	496 (24.3%)	522 (25.5%)	11.3317	0.0093
Female	578 (28.3%)	451 (22.0%)	6.8016	
**Number of children**				
1–2	515 (25.2%)	571 (27.9%)	24.8405	0.0004
3–4	332 (15.2%)	252 (12.3%)	8.0104	
5 and above	228 (11.1%)	150 (7.3%)		

Note: Where n is less than 2059, it is due to missing values

Exclusive breastfeeding is associated with the age of the mothers, ethnicity of the mother, sex of the child and the number of children the mother has borne. On the other hand, education, religion, region, employment status, wealth index and marital status of the mothers were not statistically associated with exclusive breastfeeding of babies from birth up to 6 months.

### Multivariate analysis

A binary logistic regression model was fitted including variables significant in the bivariate analysis. The results are shown in Table 5.

**Table 5:** Determinants of exclusive breastfeeding in infants of 6 months and below

**Variable**	**Odds Ratio**	***P*-value**	**95% CI**	**Overall** ***P*-value**
**Age of mothers (years)**				
15 to 24	*			0.0144
25 to 34	0.91	0.545	0.66–1.24	
35 and above	1.63	0.059	0.98–2.71	
**Ethnicity of mothers**				
Chewa	*			
Tumbuka	1.71	0.012	1.13–2.59	
Lomwe	1.01	0.968	0.74–1.36	0.0195
Tonga	1.18	0.563	0.67–2.08	
Yao	1.22	0.262	0.86–1.72	
Sena	0.99	0.968	0.58–1.69	
Nkhonde	1.76	0.218	0.71–4.35	
Ngoni	2.05	< 0.001	1.38–3.05	
Mang’anja	1.07	0.838	0.54–2.13	
Nyanja	1.14	0.819	0.38–3.40	
Other	0.80	0.495	0.42–1.52	
**Sex of Infants**				
Male	*			
Female	1.35	0.009	1.08–1.70	0.0091
**Number of siblings**				
1–2	*			
3–4	1.47	0.028	1.04–2.08	0.0815
5 and above	1.41	0.135	0.90–2.20	

Note: *: Reference category, *C.I* Confidence interval

### Age of the mother

The odds of exclusively breastfeeding for a mother aged 25 to 34 years is 0.91 times high compared to the reference group of mothers aged 15 to 24 years. This category is not significant on the outcome variable. Similarly, the odds of exclusively breastfeeding for a mother aged 35 years and above is 1.63 times as high as the odds of mothers aged 15 to 24 years being exclusively breastfeeding. This category is also not significant on the outcome variable.

### Ethnicity of the mother

The above results indicate that only Tumbuka and Ngoni are significant on the outcome variable. The odds of exclusively breastfeeding for a mother who is a Tumbuka is 1.71 and Ngoni is 2.05 times as high as the odds of exclusively breastfeeding for a mother who is of Chewa tribe (reference group).

### Sex of infant

Compared to the reference group (male child), the odds of exclusively breastfeeding a female child is 1.35 times as high with confidence interval of 1.08 to 1.70 and overall *P*-value of 0.0091.

### Number of children of the mother

In terms of the number of children of the mother, only mothers having 3 to 4 children were significant on the outcome variable. The odds of exclusively breastfeeding for a mother having 3 to 4 children is 1.47 times high compared to the reference group of a mother having 1 to 2 children.

## Discussion

This study assessed determinants of exclusive breastfeeding in children of 6 months of age in Malawi using cross–sectional household MDHS data collected in 2015–2016. In the MDHS report, it was indicated that the prevalence of EBF was 61.4%. However, the current study reveals that the prevalence of EBF is 51.4%. This discrepancy came about because MDHS analyzed data from women with children under 6 months of age, while this analysis used data for women with children who completed 6 months from birth.

We find that EBF declines with age; this trend has been consistent in different studies. In Tanzania, Ethiopia and Nigeria, the prevalence of EBF was found to be decreasing with increase in age [3, 5, 13]. The reason for this trend mostly has been that mothers perceive that breast milk alone would not be enough for the perceived demands of the growing child.

MDHS defined EBF as giving baby only breastmilk for the first 6 months of life. Based on this definition, the study identified four factors to be associated with exclusive breastfeeding in Malawi. These factors are age of the mother, ethnicity of the mother, sex of the infant and number of children of the mother.

Usually, an adolescent mother is considered less likely to continue EBF in comparison to older women due to the higher likelihood of young women being single and due to the urgency of attending school [14]. By contrast, we did not observe a relationship between individual age bands of the mother and EBF. However, age of the mother on exclusive breastfeeding is significant. This association has been noted in Western countries [15].

In Quito, Ecuador, about 62.9% of adolescent mothers exclusively breastfed their infants within the first 6 months of life [14]. This rate is higher than the one estimated for all mothers in Ecuador (43.8%) [16] and higher than the EBF prevalence reported in other countries among adolescent mothers, ranging from 52% in the United States of America to 13.8% in Brazil [17].

In the present study, mothers who are Tumbukas and Ngonis were more likely to practice exclusive breastfeeding as compared to other ethnic groups. Cultural factors are thus at play even within a country: between countries other cultural variations have been noted, such as giving water plus breast milk by some communities in Nigeria to quench the child’s thirst [5]. In Ghana, it was also reported that the low practice of exclusive breastfeeding in all regions could be attributed to cultural beliefs [18]. Mothers or relatives usually give water and other concoctions to infants as a perceived way of quenching their thirst or as a sign of welcoming them into the world [19].

In Nairobi, Kenya, all other ethnic groups apart from the Kamba were more likely to stop breastfeeding their infants compared to Kikuyu women [20]. There is no established reason for this but it could be multi-factorial, including cultural practices related to breastfeeding and child rearing like giving babies herbals to boost their immunity.

It has been argued that HIV prevalence would lead to early cessation of EBF [20]. Mothers who are HIV positive are more likely to stop exclusive breastfeeding. In Malawi, HIV prevalence is low in the northern region (where Tumbukas and Ngonis are largely found) than southern and central [7]. This has been attested by MPHIA Survey of 2015–2016 which reported that overall prevalence of HIV in northern region was 7.4%, central region 22.5% and in southern region 49% [21]. Therefore, this could partially explain why Tumbukas and Ngonis might practice EBF more than others, despite the strong emphasis in public health messaging about EBF in the context of prevention of maternal to child transmission of HIV.

Mothers with female infants have higher odds of practicing EBF compared to those with boys. Evidence from different studies in Nigeria agrees that sex of the baby significantly affects the rate of EBF whereby female infants were more likely to be exclusively breastfed than male infants [5]. Another study in Nairobi, Kenya reports that boys were more likely to be introduced to complementary feeding early compared to girls [19]. It was further argued that anecdotal evidence indicated that boys are introduced to complementary foods early because breast milk alone does not meet their feeding demands, which could be the same case with Malawi.

In the current study, the number of children was statistically significant in predicting the rate of exclusive breastfeeding the infants of 6 months in Malawi. Mothers having 3 to 4 children were more likely to breastfeed their children compared to those with 1 to 2 and over 4 children. Though there are no reported studies to support this finding, it could be that the marital, family or social conditions for mothers with 3 to 4 children are more favorable in some way. Further, ethnographic research is needed to understand the social and family dynamics that might support EBF.

### Recommendation

The results reveal a markedly decreased rate of EBF with increase in the age of infants. Therefore, there is a need for targeted interventions. Community breastfeeding groups may help to maintain breastfeeding by supporting mothers with breastfeeding problems right in their communities. The “10 steps to successful breastfeeding” program implemented under the Baby Friendly Hospital Initiative (BFHI) program may be ripe for re-examination and revival in the Malawi setting.

Health education and awareness campaigns to sensitize communities on implications of some cultural practices on the lives of babies need to be intensified. Other interventions could be the use of mass media, targeted health surveillance assistants’ home visits and strengthening of male involvement through appreciating the importance of EBF and extending support to partners to maintain this.

However, intervention studies need to be designed and formally tested to assess the impact of community support groups in promoting exclusive breastfeeding up to 6 months after women have been discharged from the hospital. Again, the timing of immunization schedules could be assessed to see if breastfeeding advice and support can be combined with vaccination visits.

Qualitative research could be used to investigate perceptions of mothers and their motivation in EBF. Additionally, there is need for longer term nutritional surveillance on healthy outcome of children with EBF and those without EBF.

### Study strengths and limitation

The use of cross-sectional data only allows associations to be established, but not causality. However, the key strength of this study was that it used MDHS dataset which is nationally representative, and provided reliable estimates at the national and regional levels, for urban and rural areas, and for each of the 28 districts. Additionally, appropriate adjustment for sampling design, including sampling weights was employed and there was a very high response rate (98%) to the survey interview.

## Conclusion

We identify important variations in EBF practices among population sub-groups in Malawi that need to be considered when framing health education messaging. Work is needed to assess the impact of more targeted messaging, whether delivered via ‘ten steps’ programming or other health education and awareness campaigns to sensitize communities on implications of some cultural practices on the lives of infants. The potential role for mass media, targeted Health Surveillance Assistants’ (HSA) home visits and male involvement also require exploration.

## Data Availability

The data set used was obtained from DHS Program under their access arrangements. DHS Program provides the data set upon reasonable request at https://www.dhsprogram.com/data/dataset/Malawi_Standard-DHS_2015.cfm?

## Abbreviation

BFHI: Baby Friendly Hospital Initiative
CCAP: Church of Central African Presbyterian
COMREC: College of Medicine Research Ethics Committee
EBF: Exclusive breastfeeding
HIV: Human Immunodeficiency Virus
HSA: Health Surveillance Assistant
MDG: Millennium Development Goals
MDHS: Malawi Demographic and Health Survey
MICS: Multiple Indicator Cluster Survey
MPHIA: Malawi Population-based HIV Impact Assessment
NSO: National Statistical Office
UNICEF: United Nations Children’s Fund
USAID: United States Agency for International Development
WHO: World Health Organization
