# Correction to: Determinants of exclusive breastfeeding in infants of six months and below in Malawi: a cross sectional study

**DOI:** 10.1186/s12884-020-03232-z

**Published:** 2020-09-11

**Authors:** Yusuf M. Salim, William Stones

**Affiliations:** 1 Karonga District Hospital, Private Bag 1, Karonga, Malawi; 2 College of Medicine, Private Bag 360, Blantyre, Malawi

**Correction to: BMC Pregnancy Childbirth 20, 472 (2020)**

**https://doi.org/10.1186/s12884-020-03160-y**

Following publication of the original article [1], we have been notified that under **Discussion** section on page 6 of 8, paragraph 3, there is an error. In this Correction, incorrect and correct versions are shown.

**Incorrect:** “In Malawi, HIV prevalence is low in the northern region (where Tumbukas and Ngonis are largely found) than southern and central [7]. This has been attested by MPHIA Survey of 2015–2016 which reported that overall prevalence of HIV in northern region was 7.4%, central region 22.5% and in southern region 49% [21].”

**Correct:** “In Malawi, HIV prevalence varies regionally. Northern region (where Tumbuka and Ngoni communities are largely found) records a prevalence of 7.4% with 4.9% - 6.1% in rural zones of the central region, rural zones of the southern region at 15.3 - 16.0% and as high as 17.7% in Blantyre, the main urban centre in the south [21].”
